# Analysis of the cannabinoid content of strains available in the New Jersey Medicinal Marijuana Program

**DOI:** 10.1186/s42238-019-0011-z

**Published:** 2019-12-26

**Authors:** Thomas A. Coogan

**Affiliations:** New Jersey Cannabis Industry Association, 130 West State Street, Trenton, NJ 08608 USA

**Keywords:** Cannabis strains, Testing, Medical Cannabis, Cannabinoid ratio

## Abstract

**Background:**

The six licensed operators in the New Jersey Medicinal Marijuana Program submit their strains of cannabis flower to a single laboratory, administered by the state’s Department of Health, for testing. The results of these tests are made available by the State on a web page for patients, allowing a study of the range of cannabinoid profiles available in the program.

**Methods:**

Reports on cannabinoid concentrations were collected from 245 test reports released by the State lab; the relative quantities of cannabinoids on all strains was evaluated, as well as trends in the strain types being tested.

**Results:**

The collection of strain profiles available in New Jersey conforms to results of other population studies, revealing three broad classification of strains based on their relative concentration of cannabinoids: the overwhelmingly majority of strains contain only trace (< 1%) CBDA but high THCA concentration; a handful are balanced in CBDA and THCA content; and a very few strains have a high concentration of CBDA and minimal THCA (< 1%). In those strains that contain more than 1% of both THCA and CBDA, those two substances are present in comparable quantities. The concentration of CBGA is higher in those strains that have the highest THCA concentration, though there are strains that have high THCA (> 20%) with CBGA concentrations at the low end of the range (< 0.5%). In the high CBD strains, the concentration of CBGA is positively correlated with CBDA, but the CBGA concentrations are several fold less in CBD-dominant strains than in THC-dominant strains: the highest measured CBGA concentration in a CBD-dominant strain is only at the average value of CBGA concentration in THC-dominant strains. The most-recently tested strains are overwhelmingly of the THC-dominant type.

**Conclusions:**

Though some high CBD strains are available in the New Jersey medical marijuana program, the vast majority of strains that have been tested are the THC-dominant strains which contain less than 1% CBDA. The data available from the State does not include any information on how well the different strains sell, but it can be inferred from the trend in strain types tested that the demand in the New Jersey medical market is for THC-dominant strains.

## Background

The State of New Jersey approved medical marijuana with the “New Jersey Compassionate Use Medical Marijuana Act” in 2010, based on its finding that “Modern medical research has discovered a beneficial use for marijuana in treating or alleviating the pain or other symptoms associated with certain debilitating medical conditions, as found by the National Academy of Sciences’ Institute of Medicine in March 1999”. These findings have been validated by the 2017 report by the National Academies of Sciences, Engineering, and Medicine (NationalAcademies.org/cannabishealtheffects).

The 2010 legislation established Alternative Treatment Centers (ATC) to grow, process, and dispense product under the regulation of the Department of Health (DoH). As of 2019, six ATCs were operating and had delivered 5200 pounds of product to patients in 2017 (DoH Division of Medicinal Marijuana Annual Report). The State’s Public Health and Environmental Laboratories (PHEL) and Agriculture Department lab have done quality and potency testing for the ATCs, and make data available on the strains that each ATC has produced (Alternative Treatment Center Medicinal Marijuana Strain Library, njmmp.nj.gov/njmmp/jsp/marijuanaStrainDocsForptlogin.jsp). All of the testing reported by this lab is for flower product.

Cannabis is marketed today using many colorful names for individual strains. Whether they are all properly termed strains or chemovars or simply variants might be debated, but the State of New Jersey has registered each sample that is submitted with a different name as a “strain”. The more colorful and traditional terminology, such as “indica” and “sativa” descriptors for strains, is how the products are described on the ATCs websites, with the actual cannabinoid level information only available from the DoH Web site or at the dispensary. A clearer picture of the strains offered and chosen by New Jersey patients might provide greater insight into the specific properties of medicinal marijuana that are valued.

The data provided by the Medicinal Marijuana program on the lab testing of the strains produced by NJ ATCs allows an evaluation of the relative levels of the reported cannabinoids in each strain, the number of strains offered with those profiles, and trends in potency of strains offered.

## Methods

The PHEL method for potency testing has been published (Patel et al. 2017). In short, after grinding, extraction, centrifugation, filtration and dilution, samples were analyzed by high pressure liquid chromatography (HPLC) using an Agilent Poroshell 120 SB-C18 column.

In the reports that the lab provided, they have reported results for up to 5 individual samples for each strain, and results for a composite of the five samples together. Concentrations are reported in percentage by weight. A strength of taking this data set from one laboratory which has regulatory oversight over the growers is that it provides a control for variables (e.g. sampling, processing, drying) that may exist when comparing results from a variety of labs and protocols. Reports from the State site were accessed from the Medicinal Marijuana site (https://njmmp.nj.gov/njmmp/jsp/marijuanaStrainDocsForptlogin.jsp), and data on cannabinoid profile was collected from the tests which were run on composites of five samples for each strain. The data was collected for each of the 8 cannabinoids which the lab reports on: Cannabigerolic acid (CBGA), Cannabigerol (CBG), Cannabidiol acid (CBDA), Cannabidiol (CBD), Tetrahydrocannabinol acid (THCA), Delta-9-Tetrahydrocannabinol (THC), Delta-8-Tetrahydrocannabinol, and Cannabinol (CBN).

**Fig. 1:**
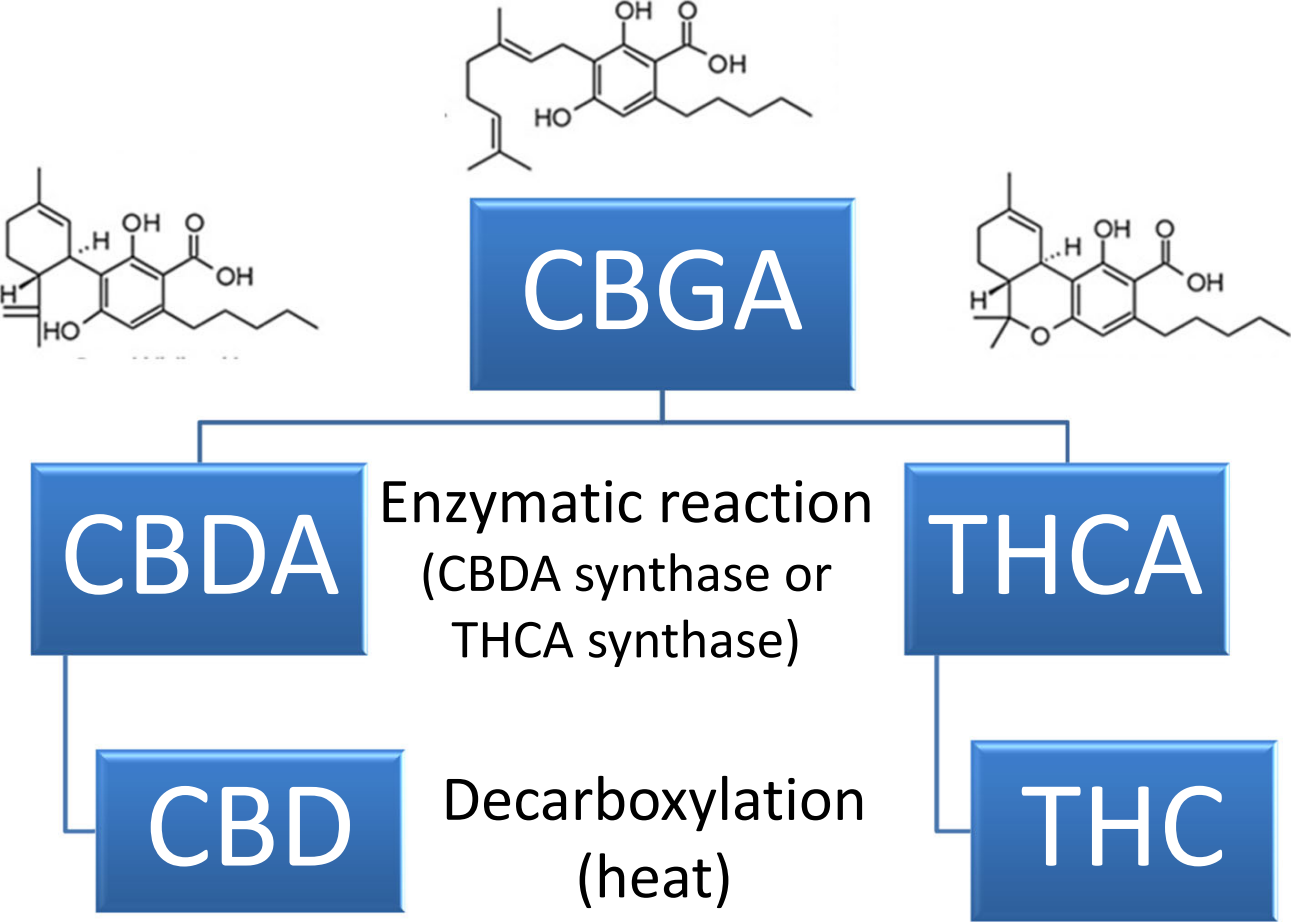
Relationships of major cannabinoids. Cannabidiol acid (CBDA) and Tetrahydrocannabinol acid (THCA) are synthesized from Cannabigerolic acid (CBGA) by enzymatic reaction. These acidic molecules are changed to the more commonly known forms by decarboxylation, usually by applying heat

The relationship among the major cannabinoids is depicted in Fig. 1: CBD and THC are formed by decarboxylation of the acidic forms of these molecules (CBDA and THCA), the form in which they are originally synthesized in the plant. CBDA and THCA are separately generated by enzymatic reactions from their common precursor, CBGA (Taura et al. 1995, 1996).

## Results

The posted reports were collected for 245 test results from the PHEL on strains produced by New Jersey ATCs, dating from 2014 through mid-2019. Not every report has entries for all 8 cannabinoids listed on the reports; on all but a few reports, a “Not Detected” entry is made for CBN and Delta-8-Tetrahydrocannabinol.

The average THCA concentration across all reports was 18.2%; the average CBDA concentration was 0.7%. In all of the strains reported on, CBGA was not found to accumulate above 2.5%.

**Fig. 2:**
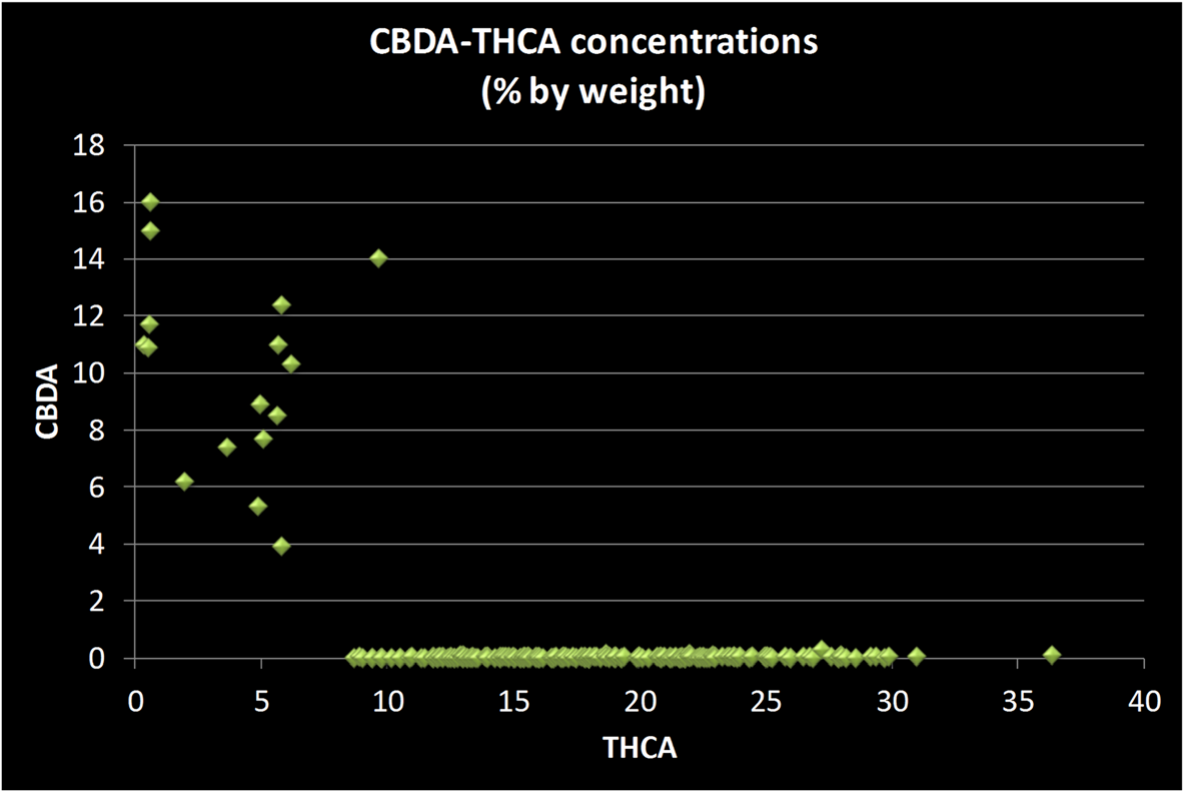
Scatter plot of CBDA versus THCA concentrations in strains sold in the New Jersey medicinal marijuana program. Each data point represents two values from a single strain: its concentration of Cannabidiol acid (CBDA) and of Tetrahydrocannabinol acid (THCA). Three clusters of strain types are evident: THC-dominant strains which have less than 1% CBDA (clustered along X-axis); balanced strains with comparable amounts of CBDA and THCA (middle of chart); and CBD-dominant strains which have less than 1% THCA (along Y-axis). The overwhelming majority of strains (229 out of 245) are THC-dominant. All measurements done by the State of New Jersey’s Public Health and Environmental Laboratories using chromatographic methods and reported as percentage by weight

The average values for cannabinoid levels do not reveal the dispersion in range of concentrations seen across the data set. This is particularly notable for CBDA concentrations; over 200 tests had CBDA values below 1%, while 15 had CBDA concentrations ranging from 5 to 16%. In those strains with significant CBDA concentrations, the THCA concentrations were well below the average THCA values for all strains. None of the strains which had CBDA concentration above 3% had a THCA concentration above 10% (Fig. 2).

**Fig. 3:**
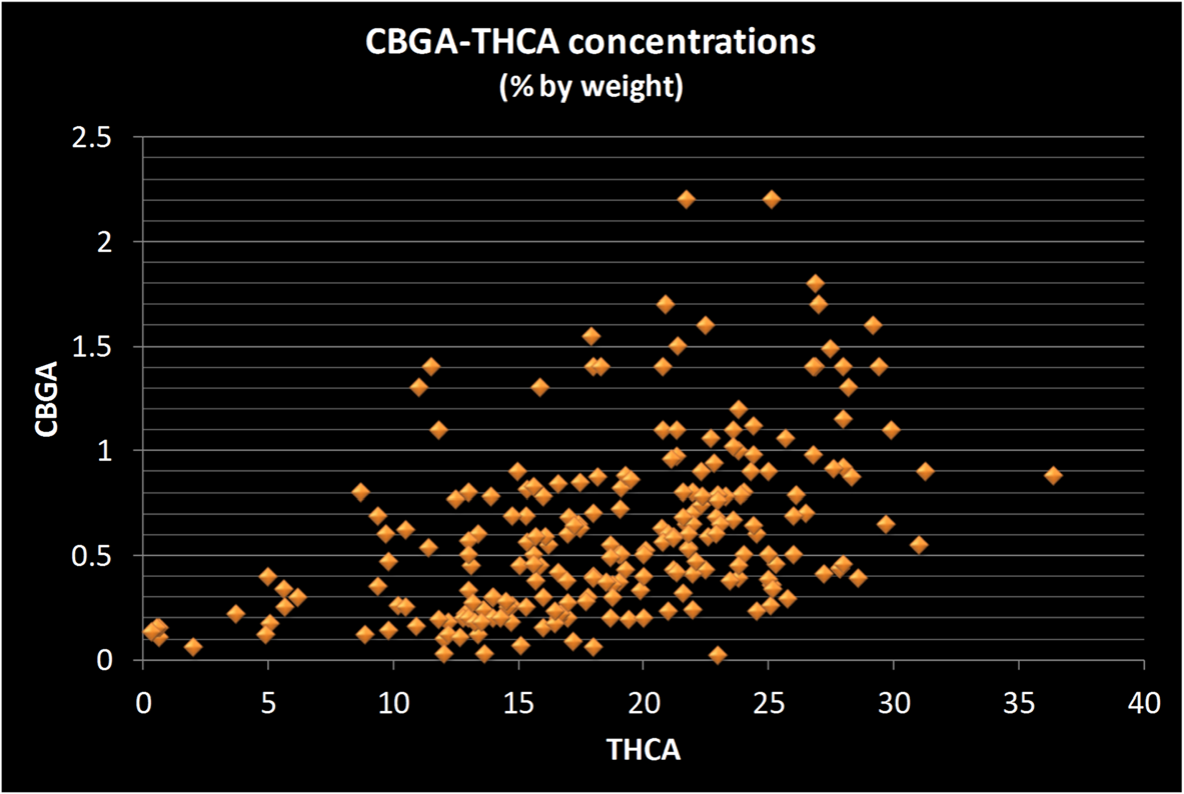
Scatter plot of CBGA versus THCA concentrations in strains sold in the New Jersey medicinal marijuana program. Each data point represents two values from a single strain: its concentration of Cannabigerolic acid (CBGA) and of Tetrahydrocannabinol acid (THCA). The strains with the highest concentration of CBGA (Y-axis) are those with on the high end of the THCA concentrations (X-axis). The relationship is not a clear one, however, as there are strains near the highest in THCA concentration (> 25%) which have only moderate CBGA concentration. All measurements done by the State of New Jersey’s Public Health and Environmental Laboratories using chromatographic methods and reported as percentage by weight

Relationships between relative amounts of other cannabinoids were analyzed graphically in scatter plots. The concentrations of THCA and of CBGA are positively correlated, as expected since CBGA is the immediate precursor in the synthesis of THCA. The precursor molecule, CBGA need not be present at high levels for the strain to have a very high (> 20%) THCA concentration, but the higher CBGA concentrations are only found in the strains with above-average THCA levels (Fig. 3).

**Fig. 4:**
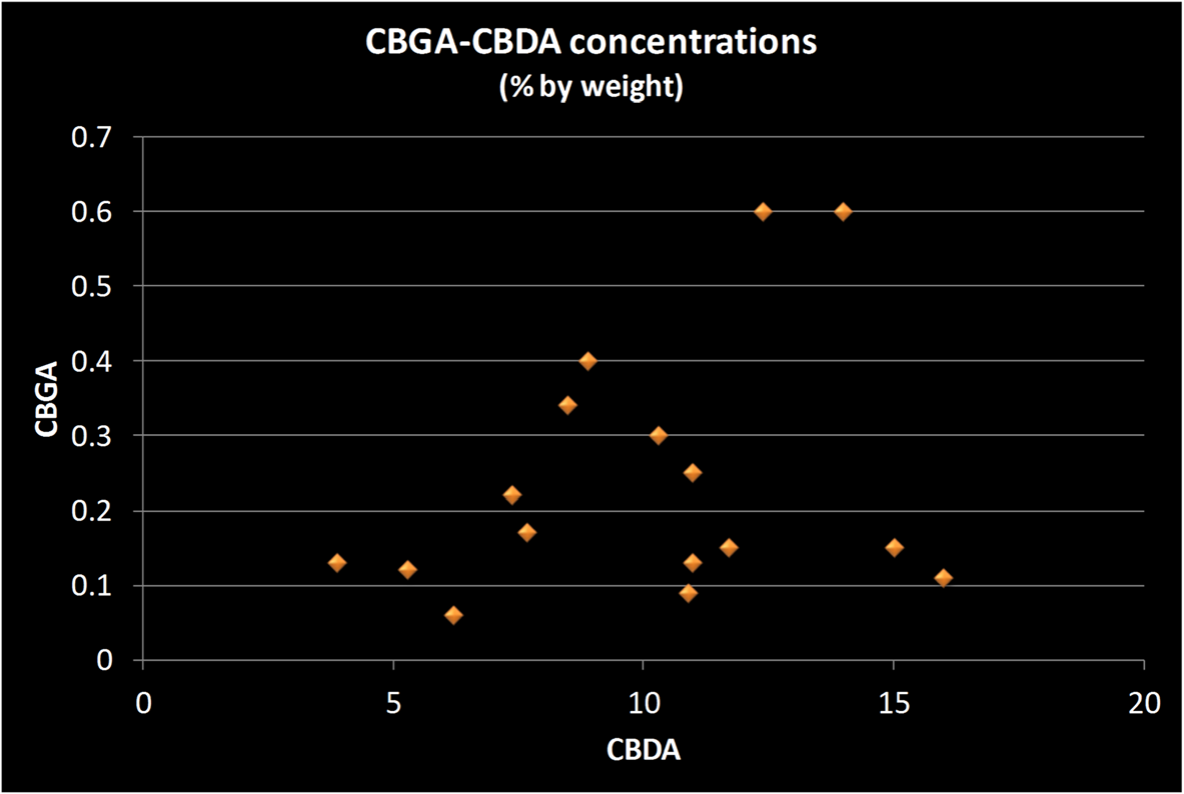
Scatter plot of CBGA versus CBDA concentrations in strains sold in the New Jersey medicinal marijuana program. Each data point represents two values from a single strain: its concentration of Cannabidiol acid (CBDA) and of Cannabigerolic acid (CBGA). The concentration of CBGA is much less in strains which are high in CBDA than in strains with are high in THCA. The plot of values for the sixteen strains which had more than 1% CBDA shows a positive relationship between the concentration of CGDA and CBGA, but no single CBGA concentration is above 0.6%, while that is the average CBGA value for THC-dominant strains. All measurements done by the State of New Jersey’s Public Health and Environmental Laboratories using chromatographic methods and reported as percentage by weight

To get a view of the CBGA-CBDA relationship, the data set was restricted to those strains which contain more than 1% CBDA, and find a similar positive relationship is seen between CBGA and CBDA concentrations (Fig. 4). The CBGA concentration in high CBDA strains is notably lower (< 1%) than the highest values seen in high THCA strains. The average CBGA concentration in CBD-dominant strains is 0.24%, while it is 0.61% in THC dominant strains; more than 30 THC-dominant strains have CBGA values greater than 1%.

**Fig. 5:**
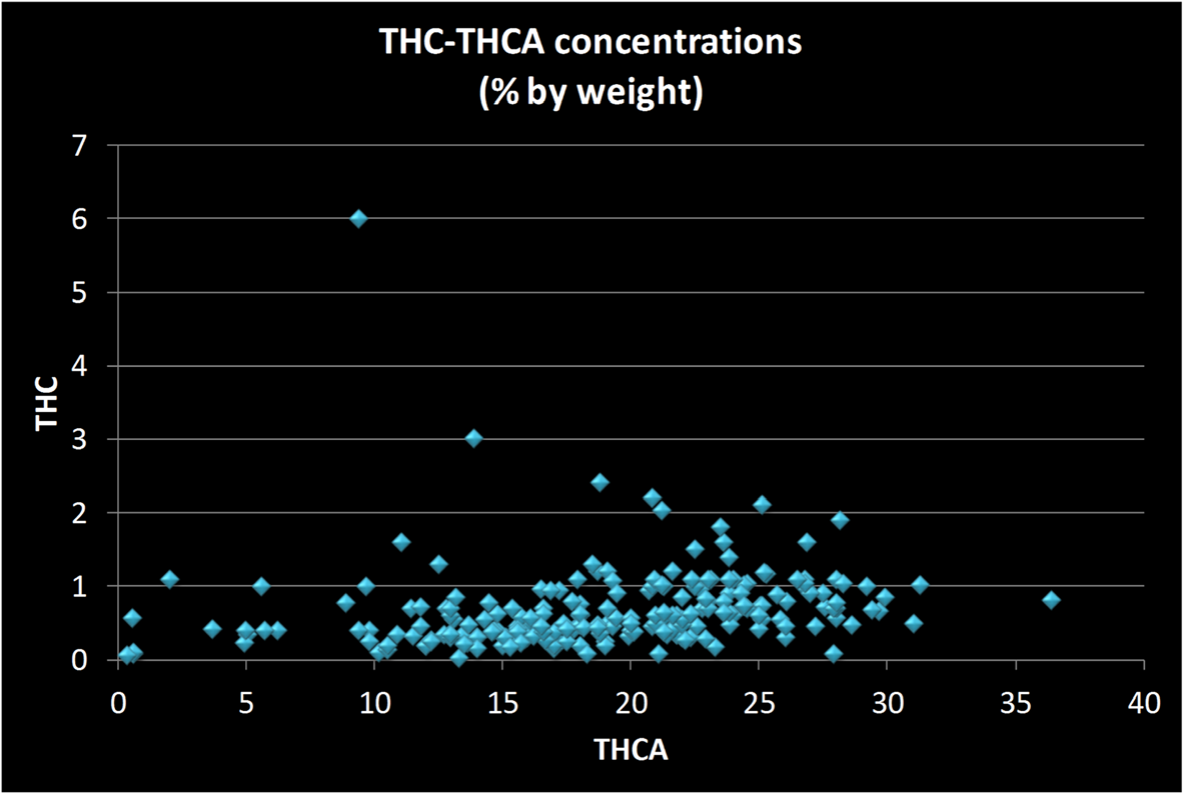
Scatter plot of THC versus THCA concentrations in strains sold in the New Jersey medicinal marijuana program. Each data point represents two values from a single strain: its concentration of Tetrahydrocannabinol (THC) and of Tetrahydrocannabinol acid (THCA). Some THC does accumulate in flower product, and in higher levels in strains that have the highest THCA concentration, though the relationship is not a liner one. In those strains in which the levels do accumulate in sufficient quantity, that quantity should be taken into account when characterizing the full cannabinoid content of strain. All measurements done by the State of New Jersey’s Public Health and Environmental Laboratories using chromatographic methods and reported as percentage by weight

Though most consumers speak of THC concentration, native flower contains very little of this substance, compared to THCA (Fig. 5). The small variations seen in the THC concentrations across strains is not well correlated with THCA levels and may have more to do with product handling than with differences in plant physiology.

The trend in strain types tested was evaluated by separately analyzing just those results posted for the most recent 6 months (79 strains). There is a trend of increasing concentration evidenced by the average THCA concentration for the recent 6 months being higher (22.5%) than the value for the 166 prior entries (16.2%). Additionally, of the recent 79 strains, only one has a CBDA concentration above 1%.

## Discussion

Using data made available by the State of New Jersey’s testing lab, I have analyzed the cannabinoid content of the strains that have been offered in the state’s medical marijuana program. This analysis is limited to the data which the State has made available, so it does not include any information on the relative sales volume of the different strains, nor any discrimination between strains based on their terpene content. Each of the strains offered by New Jersey’s ATCs may be quite distinct, and uniquely valued by patients, for properties other than those analyzed and reported by the PHEL.

The legislation that originally authorized New Jersey’s medicinal marijuana program stipulated that each of the ATCs were restricted to only offering three strains and be of high, medium, or low strength, to encourage the inclusion of high CBD strains. In 2013, the governing legislation was amended to remove constraints on the ATCs in terms of the strain types carried. Today 4 of the 6 ATCs offer a high CBD strain, but with few exceptions, the new strains being tested have negligible CBDA content. The interests that the State had in the policy of requiring strains of different strengths might be addressed by requiring a minimum range of strain strengths, while not imposing the burden of restricting the growers to *only* 3 strains.

The CBGA molecule is the precursor for both CBDA and THCA, and is also of some interest for possible therapeutic properties of CBG itself (Borrellia et al. 2013; Granja et al. 2012). Whether the values of CBGA found in these samples (below 3%) would be a physiologically significant amount after human consumption depends on what the receptor affinity is for that molecule, which has not been established. This point is even more critical for CBD-dominant strains, which in the New Jersey sample have significantly less CBGA present than in THC-dominant strains. Whether this is a general trait found in strains other than those in the New Jersey program, is a question that should be investigated in larger samples. Support for this observation may be found in a recently published analysis of 161 Italian hemp samples (Palmieri et al. 2019). That study reported low levels of CBG and CBGA in all samples, with the exception of samples from one retailer that had higher THCA concentrations than the samples from other retailers. Since there are relatively few high CBD strains in the New Jeresy set, it is important to point out that there are THC-dominant strains with CBGA levels as low as those in the CBD-dominant strains; testing a greater variety of CBD-dominant strains may reveal that there are some with CBGA levels as high as found in some THC-dominant strains.

While CBGA concentrations are several-fold lower than THCA concentrations in every strain, there is a positive relationship between the two, though not clearly linear. The precursor molecule, CBGA, need not be present at high levels for the strain to have a very high (> 20%) THCA concentration, but the higher CBGA concentrations are found in the strains with above average THCA levels. One interpretation of this is that excess CBGA is only accumulated as the plant reaches a limit for THCA production.

Scatter plots of THCA against CBDA concentrations suggest three broad groups of strains:

- those with < 1% CBDA and with THCA concentration from 10 to 30%;
- those with both THCA and CBDA concentrations in the 5–10% range; and
- those with < 1% THCA and with CBDA concentration > 10%.

This clustering of strains is similar to other published studies of strain collection (Hillig and Mahlberg 2004; Jin et al. 2017; Jikomes and Zoorob 2018), and reflects the propagation of strains which lack a functional gene for CBDA synthase, the enzyme which forms CBDA from CBGA (de Meijer et al. 2003). In strains in which the CBDA synthase enzyme is not converting CBGA into CBDA, higher levels of THCA can be accumulated.

A striking feature of the plots of CBDA concentrations against THCA concentrations is the positive correlation between the two for those strains which make each compound in significant quantity (the center cluster). The finding that all those strains with high THCA also have corresponding high CBDA levels, in that subset of strains, is also a striking finding of other studies of a range of strain collections, from the Canadian medical market (Jin et al. 2017), a collection of strains from diverse sources including breeders and law enforcement (Hillig and Mahlberg 2004) and a study of the legalized market in the State of Washington (Jikomes and Zoorob 2018). Judging from these studies, it appears to be very uncommon to have strains that would have more than 15% THCA and have more than a negligible amount of CBDA (> 3%). This suggests that growers face a choice: strains that have both THCA and CBDA in roughly equal amounts; or give up on getting any significant CBDA concentration to reach double digit THCA concentrations.

The trend in strains being introduced into the New Jersey medical market is toward higher THC content. The data available from the State’s patient portal do not show relative sale volumes for the different strains, so we cannot determine if there is a potency trend in the product which is actually sold and consumed. It must also be pointed out that the State lab only tests flower product, and two New Jersey ATCs have extract products available. It is a reasonable supposition, though, that the strains chosen for development and commercialization by the ATCs reflect customer demand for high THC strains over those with significant CBD levels.

## Conclusions

Strains produced for New Jersey’s medical marijuana program fall into expected clusters based on cannabinoid levels: THC-dominant strains (< 1% CBDA); balanced strains; and CBD-dominant strains (< 1% THCA). In balanced strains, THCA and CBDA are produced in proportional amounts. New Jersey’s licensed producers test THC-dominant strains much more frequently than balanced or CBD-dominant strains. CBGA levels are correlated with THCA and CBDA levels, and are on average higher in strains that are high in THCA than in strains high in CBDA. The constraints on which combinations of cannabinoids can be produced at high levels have important implications for therapeutic use of flower products.

## Data Availability

The strain test results for New Jersey’s medical program are available in the Department of Health’s Alternative Treatment Center Medicinal Marijuana Strain Library: njmmp.nj.gov/njmmp/jsp/marijuanaStrainDocsForptlogin.jsp. To access files, click on the “Marijuana Strain Lib” tab at top left, then the use pull down menus for the individual ATCs.

## Abbreviations

ATC: Alternative Treatment Center
CBDA: Cannabidiol acid
CBGA: Cannabigerolic acid
DoH: Department of Health
HPLC: High pressure liquid chromatography
PHEL: Public Health and Environmental Laboratories
THC: Delta-9-Tetrahydrocannabinol
THCA: Tetrahydrocannabinol acid
